# Use of public sector diabetes eye services in New Zealand 2006–2019: Analysis of national routinely collected datasets

**DOI:** 10.1371/journal.pone.0285904

**Published:** 2023-05-18

**Authors:** Pushkar Raj Silwal, Arier C. Lee, David Squirrell, Jinfeng Zhao, Matire Harwood, Andrea L. Vincent, Rinki Murphy, Shanthi Ameratunga, Jacqueline Ramke

**Affiliations:** 1 School of Optometry and Vision Science, Faculty of Medical and Health Sciences, University of Auckland, Auckland, New Zealand; 2 School of Population Health, Faculty of Medical and Health Sciences, University of Auckland, Auckland, New Zealand; 3 Eye Department, Greenlane Clinical Centre, Auckland District Health Board, Auckland, New Zealand; 4 Department of Ophthalmology, Faculty of Medical and Health Sciences, New Zealand National Eye Centre, University of Auckland, Auckland, New Zealand; 5 Department of General Practice and Primary Care, Faculty of Medical and Health Sciences, University of Auckland, Auckland, New Zealand; 6 Auckland Diabetes Centre, Greenlane Clinical Centre, Auckland District Health Board, Auckland, New Zealand; 7 Whitiora Diabetes Service, Middlemore Hospital, Counties Manukau Health, Auckland, New Zealand; 8 Department of Medicine, Faculty of Medical and Health Sciences, University of Auckland, Auckland, New Zealand; 9 Population Health Directorate, Counties Manukau Health, Auckland, New Zealand; 10 International Centre for Eye Health, London School of Hygiene & Tropical Medicine, London, United Kingdom; Auckland University of Technology, NEW ZEALAND

## Abstract

**Objective:**

To assess diabetes eye service use in New Zealand among people aged ≥15 years by estimating service attendance, biennial screening rate, and disparities in the use of screening and treatment services.

**Methods:**

We obtained Ministry of Health data from the National Non-Admitted Patient Collection on diabetes eye service events between 1 July 2006 and 31 December 2019 and sociodemographic and mortality data from the Virtual Diabetes Register and linked these using a unique patient identifier (encrypted National Health Index). We 1) summarized attendance at retinal screening and ophthalmology services, 2) calculated biennial and triennial screening rate, 3) summarized treatment with laser and anti-VEGF and used log-binomial regression to examine associations of all of these with age group, ethnicity, and area-level deprivation.

**Results:**

In total, 245,844 people aged ≥15 years had at least one diabetes eye service appointment attended or scheduled; half of these (n = 125,821, 51.2%) attended only retinal screening, one-sixth attended only ophthalmology (n = 35,883, 14.6%) and one-third attended both (n = 78,300, 31.8%). The biennial retinal screening rate was 62.1%, with large regional variation (73.9% in Southern District to 29.2% in West Coast). Compared with NZ Europeans, Māori were approximately twice as likely to never receive diabetes eye care or to access ophthalmology when referred from retinal screening, 9% relatively less likely to receive biennial screening and received the fewest anti-VEGF injections when treatment was commenced. Disparities in service access were also present for Pacific Peoples compared to NZ Europeans, younger and older age groups compared to those aged 50–59 years and those living in areas with higher deprivation.

**Conclusions:**

Access to diabetes eye care is suboptimal, with substantial disparity between age groups, ethnicity groups, area level deprivation quintile and across districts. Efforts to improve access to and quality of diabetes eye care services must include strengthening data collection and monitoring.

## Introduction

Diabetic retinopathy (DR) is the most common microvascular complication of diabetes and is the leading cause of blindness among the working-age population globally [1]. Almost everyone with Type 1 diabetes and >60% with Type 2 diabetes will develop some degree of DR after 20 years of diabetes [2]. Major risk factors for DR include poor glycaemic control, hypertension, smoking, renal dysfunction and dyslipidaemia [2, 3]. The risk of vision loss from DR can be reduced through good glycaemic, cholesterol and blood pressure management [4].

DR screening is a health intervention that can detect retinal changes before symptoms commence. If sight-threatening DR develops, treatment includes timely laser photocoagulation or injections of anti-vascular endothelial growth factor (anti-VEGF) [5] alongside consistent effort to improve other modifiable risk factors. If administered at an appropriate stage, early treatment can help slow disease progression [6].

In 2020, an estimated 277,800 people in Aotearoa New Zealand (hereafter referred to as New Zealand) had diabetes [7]; the Ministry of Health estimates that 20–25 percent of people with diabetes have some form of DR [8]. The New Zealand Ministry of Health has a diabetes retinal screening guideline to inform and guide the delivery of services that provide timely detection and appropriate management for people with DR [9]. The guideline recommends that 1) people with Type 1 diabetes are screened when 10 years old or five years after diagnosis, whichever occurs first, 2) people with Type 2 diabetes are screened at the time of diagnosis and 3) women with gestational diabetes are screened during the first trimester. Rescreening for those with no DR is recommended every 2 years (Type 1) or every 2–3 years depending on the presence of any clinical modifiers such as poor glycaemic control (Type 2) [9]. When moderate DR develops, people are referred from retinal screening to an ophthalmology service for closer surveillance and, if necessary, treatment such as laser or anti-VEGF injections. As a result of treatment, the DR of some will stabilise sufficiently for them to be referred back to retinal screening services, but others with more severe disease may be retained under the care of ophthalmologists [10].

Until mid-2022, each of New Zealand’s 20 District Health Boards (DHB) was responsible to provide or fund the provision of DR screening services in their district with no out-of-pocket cost to people with diabetes. The DHBs implemented a range of service models, including primarily through optometry [11] or ophthalmology clinics [12] and some delivered services in community clinics throughout the region [13].

The Ministry of Health guideline states that at least 90% of the population with diabetes should be screened at the appropriate frequency [9]. A recent systematic review of DR in New Zealand identified studies from only a small number of geographic areas, and found no reports of the population level coverage of DR services. Further, the review highlighted that access to retinal screening tended to be lower among Māori, Pacific peoples and Asians compared to NZ Europeans [14], which puts these underserved groups at increased risk of vision loss [15].

Achieving equity in health outcomes for all New Zealanders is one of four priority work programs for the current New Zealand government [16]. Two key foci of the program are to meet the rights of Māori as Indigenous peoples of New Zealand and partners in Te Tiriti o Waitangi [17]; and to improve the wellbeing of the most under-served New Zealanders i.e., Māori (who made up 16.5% of the New Zealand population in 2018) [18], Pacific people (8.1% of the population in 2018 [18]) and people living with socioeconomic disadvantage [16].

This study aims to summarise diabetes eye service use in New Zealand among people aged ≥15 years by using national-level data from public-funded services. We analysed routinely collected administrative data to report i) diabetes eye service use (being retinal screening and ophthalmology services when required) in New Zealand among people aged ≥15 years, and disparities across population subgroups; ii) attendance at biennial screening; and iii) treatment for sight threatening DR across age groups, ethnicity groups, area-level deprivation quintiles and DHBs.

## Materials and methods

This is a retrospective cohort study of New Zealanders who had at least one Ministry of Health-funded diabetes eye service appointment scheduled or attended between 1 July 2006 and 31 December 2019 and were aged 15 years or above at the time of their first appointment. Ethical approval for this study was obtained from the University of Auckland Human Participants Ethics Committee (Ref: UAHPEC 020545). Individual consent was not possible due to the analysis being retrospective and on de-identified data. All data were fully anonymized before we accessed them. We have reported the study in accordance with the RECORD statement (REporting of studies Conducted using Observational Routinely-collected Data) [19].

### Data source

The Ministry of Health provided data from two datasets—the National Non-Admitted Patient Collection (NNAPC) [20] and the Virtual Diabetes Register [7]. The NNAPC provides nationally consistent data on non-admitted (outpatient and emergency department) patient activity/services that are linked to ‘purchase units’ which the Ministry of Health uses to quantify and value medical, surgical or emergency service events [21]. Data were submitted by DHBs to the Ministry of Health and included the date, facility and type of service for each event, along with the National Health Index (a unique identifier code) of the patient [20].

The Virtual Diabetes Register is a database of people suspected as having diabetes. It uses an algorithm based on the use of diabetes related health services to identify people suspected as having diabetes, including hospital inpatient and outpatient services, laboratory tests, and pharmaceutical dispensing [7]. The database contains information about the people suspected to have diabetes, who were alive and enrolled in primary care on 31 December each year. Each year, the Ministry of Health uses data from the Virtual Diabetes Register to calculate national and regional diabetes prevalence estimates.

### Study population

The study population consisted of all individuals with at least one diabetes eye service event in the NNAPC between 1 July 2006 and 31 December 2019 (i.e., appointment scheduled and/or attended for retinal screening [code M20007] or ophthalmology services [code S40002 = 1^st^ attendance, S40003 = subsequent attendance]) at which time they were aged 15 years or above (chosen due to the higher likelihood of people <15 years having Type 1 diabetes, with a delayed first screening recommendation). The encrypted National Health Index of these individuals was used to link their diabetes eye service data from the NNAPC with their demographic data from the Virtual Diabetes Register (i.e., age, gender, ethnicity, deprivation quintile, DHB area of domicile, date of death). All individuals were able to be linked across the two datasets. Individuals were followed until death or the study end date (December 31, 2019) whichever occurred first.

The Ministry of Health advised that six of the 20 DHBs (Bay of Plenty, Hawke’s Bay, Lakes, MidCentral, Hutt Valley, and Wairarapa) stopped providing data to the Ministry prior to or during the study period. These DHBs with incomplete data were excluded from the analysis.

### Outcome variables

We assessed the provision of diabetes eye services in three ways—attendance at service appointments, attendance at biennial retinal screening, and treatment events (laser and/or anti-VEGF). For these we used the codes outlined above along with the date (day/month/year) and attendance status (ATTN) of each event.

*Attendance at service appointments* during the study period for each person was categorised as one of:

- did not attend any scheduled screening or ophthalmology appointment;
- attended only retinal screening (no ophthalmology appointment scheduled);
- attended both retinal screening and ophthalmology service;
- attended only ophthalmology service (no retinal screening appointment scheduled); or
- attended retinal screening and did not attend an ophthalmology appointment.

*Attendance at biennial screening* was defined as the number of people who attended all of their expected biennial screening appointments as a proportion of the eligible population. The eligible population included all individuals who had at least one retinal screening appointment between 1 July 2006 and 1 January 2018, lived at least 24 months from the date of the first screening appointment and were not immediately referred to ophthalmology on the first screening attendance (i.e. the DR screening date was not the same as the first appointment at ophthalmology). The follow-up start date was the date of the first screening event attended and the end date was whichever occurred first of: i) a scheduled appointment at ophthalmology (taken as the exit from retinal screening), ii) the date of death or iii) 31 December 2019 (end of the study period). The number of expected appointments with respect to the length of follow-up time was calculated for each individual based on one retinal screening event in each 26-month period after their first event. The 26-month period allowed an additional two months for the appointment to be scheduled beyond the two-year guideline from the Ministry of Health [9]. We then identified those individuals who had attended all of their expected appointments.

*Treatment (with laser and/or anti-VEGF)*: was defined as the proportion of the sample attending at least one service event who had received:

- at least one dose of laser; and/or
- at least one dose of anti-VEGF (among the subset of the sample accessing services from 2014, when anti-VEGF became available);

Among people receiving anti-VEGF treatment, we also calculated the median number of treatment events per person in the first year (within 365 days) from the date of the first dose.

### Explanatory variables

The explanatory variables obtained from the Virtual Diabetes Register were *age* (categorised as 15–29, 30–39, 40–49, 50–59, 60–69, 70–79, 80+ years); *sex* (female, male or unspecified), *prioritised ethnicity* (categorised as NZ European, Māori, Pacific, Asian, Other) [22]; *area level deprivation* (based on NZDep13 [23], mapped from the domicile code on the National Health Index) arranged into quintiles from least deprived (Q1) to most deprived (Q5); and *DHB of domicile* (mapped from the domicile code on the National Health Index).

### Analysis

Descriptive statistics were reported for patient characteristics. Counts and percentages were used for categorical data; medians and inter-quartile range (IQR) were reported for continuous variables.

Log-binomial regression was used to analyse the relationship between the binary outcome variables and the explanatory variables. Relative Risks (RR) and their 95% confidence intervals were reported. Models were adjusted for baseline variables including age, sex, ethnicity, and area level deprivation in quintiles. All tests were two sided with 5% significance level. Missing data imputation was not conducted because the number of missingness in only one of the explanatory variables (deprivation) was low (n = 399 in total and 261 in the analysis of attendance at biennial retinal screening). The cases with missing area-level deprivation data were included in the descriptive analysis but excluded in the regression models.

The distribution of attendance at biennial screening across DHB and ethnicity groups was explored. Records of people where the DHB is unspecified were excluded from this analysis (n = 236). A cartogram, which is a value-by-area visualisation technique, and a complementary geographical map with a bar plot legend were applied to represent the distribution of attendance rates. In the cartogram, the size of DHB areas is proportionate to the total number of patients who attended the biennial screening, making densely populated urban DHBs, which have small land areas, more visible.

As a form of sensitivity analysis, we explored the effect on our results of the Ministry of Health guideline of screening to occur every “two to three years” [9] by repeating our calculation for the screening attendance, using 38 months (3 years plus 2 months) instead of 26 months (2 years plus 2 months). For this analysis we used the subset of people who attended retinal screening at least once before January 1, 2017 and lived at least 36 months following their first appointment.

## Results

**Fig 1:**
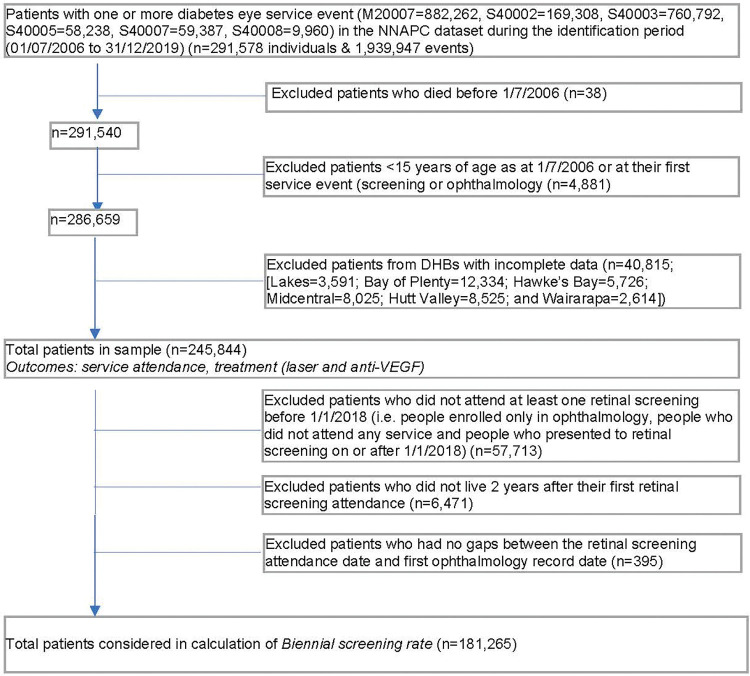
Summary of study population selection for each outcome, diabetes eye care service use in New Zealand, 2006–2019.

A total of 291,578 people had at least one diabetes eye service appointment scheduled or attended in the NNAPC between 1 July 2006 and 31 December 2019. After excluding patients <15 years of age and DHBs with incomplete data, the sample consisted of 245,844 people (Fig 1) with a median follow-up of 7.5 years (IQR 4.0–10.4). Of these, the median age was 55.5 years (IQR 44.1–66.2), 52.7% were male, 54.1% were NZ European and the proportion of participants increased with area level deprivation, from 13.4% in the least deprived quintile, up to 30.0% in the most deprived quintile (Table 1).

**Table 1:** Diabetes eye care service use and predictors of being unable to access diabetes eye care services in New Zealand, 2006–2019.

		Summary of diabetes eye care service use					Predictors of being unable to access care			
Population Subgroup	Total†	Attended DRS only	Attended DRS and ophthalmology	Attended ophthalmology only	Attended DRS but not ophthalmology when referred	Never attended DRS or ophthalmology	Never attended DRS or ophthalmology	Attended DRS but not ophthalmology when referred
	n (col%)	n (row%)	n (row%)	n (row%)	n (row%)	n (row%)	Relative Risk (95% CI)	p-value‡	Relative Risk (95% CI)	p-value‡
**Total**	**245844(100.0)**	**125821(51.2)**	**78300(31.8)**	**35883(14.6)**	**2039(0.8)**	**3801(1.5)**				
**Sex**										
Female	116292(47.3)	56515(48.6)	38269(32.9)	18809(16.2)	966(0.8)	1733(1.5)	Ref		Ref	
Male	129550(52.7)	69304(53.5)	40031(30.9)	17074(13.2)	1073(0.8)	2068(1.6)	1.16(1.08, 1.23)	<0.0001	1.04(0.95, 1.13)	0.3933
Not specified	2(0.0)	2(100.0)	0(0.0)	0(0.0)	0(0.0)	0(0.0)				
**Age (years)**										
15–29	15938(6.5)	10440(65.5)	2950(18.5)	1664(10.4)	188(1.2)	696(4.4)	3.85(3.46, 4.28)	<0.0001	1.31(1.11, 1.55)	0.0017
30–39	27579(11.2)	18761(68.0)	5658(20.5)	2100(7.6)	334(1.2)	726(2.6)	2.28(2.05, 2.54)	<0.0001	1.34(1.17, 1.54)	<0.0001
40–49	48526(19.7)	31000(63.9)	12093(24.9)	4149(8.6)	453(0.9)	831(1.7)	1.53(1.38, 1.69)	<0.0001	1.06(0.94, 1.21)	0.3406
50–59	58721(23.9)	31880(54.3)	18911(32.2)	6821(11.6)	491(0.8)	618(1.1)	Ref		Ref	
60–69	51980(21.1)	21176(40.7)	21262(40.9)	8815(17.0)	326(0.6)	401(0.8)	0.78(0.69, 0.88)	0.0001	0.79(0.68, 0.90)	<0.0001
70–79	32976(13.4)	9855(29.9)	14251(43.2)	8349(25.3)	185(0.6)	336(1.0)	1.09(0.96, 1.25)	0.1920	0.73(0.62, 0.87)	<0.0001
80+	10124(4.1)	2709(26.8)	3175(31.4)	3985(39.4)	62(0.6)	193(1.9)	2.18(1.85, 2.57)	<0.0001	0.84(0.64, 1.09)	0.1913
**Ethnicity**										
NZ European	132907(54.1)	66029(49.7)	43184(32.5)	21347(16.1)	869(0.7)	1478(1.1)	Ref		Ref	
Māori	35176(14.3)	19027(54.1)	10676(30.4)	3743(10.6)	620(1.8)	1110(3.2)	2.12(1.95, 2.31)	<0.0001	1.98(1.77, 2.22)	<0.0001
Pacific	35216(14.3)	17314(49.2)	11708(33.2)	5060(14.4)	366(1.0)	768(2.2)	1.41(1.28, 1.55)	<0.0001	1.11(0.98, 1.27)	0.1115
Asian	36795(15.0)	20367(55.4)	11153(30.3)	4802(13.1)	148(0.4)	325(0.9)	0.67(0.59, 0.76)	<0.0001	0.55(0.46, 0.65)	<0.0001
Others	5750(2.3)	3084(53.6)	1579(27.5)	931(16.2)	36(0.6)	120(2.1)	1.58(1.31, 1.90)	<0.0001	0.84(0.60, 1.18)	0.3149
**Area level deprivation (quintiles)**								
1 Least deprived	32960(13.4)	18594(56.4)	9232(28.0)	4650(14.1)	164(0.5)	320(1.0)	Ref		Ref	
2	38658(15.7)	20440(52.9)	11930(30.9)	5696(14.7)	185(0.5)	407(1.1)	1.06(0.92, 1.23)	0.4020	0.96(0.78, 1.18)	0.6764
3	44848(18.2)	22552(50.3)	14812(33.0)	6694(14.9)	258(0.6)	532(1.2)	1.14(1.00, 1.31)	0.0580	1.09(0.90, 1.33)	0.3895
4	55212(22.5)	27455(49.7)	17940(32.5)	8366(15.2)	519(0.9)	932(1.7)	1.50(1.32, 1.70)	<0.0001	1.68(1.41, 2.01)	<0.0001
5 Most deprived	73767(30.0)	36598(49.6)	24292(32.9)	10377(14.1)	911(1.2)	1589(2.2)	1.55(1.36, 1.75)	<0.0001	1.87(1.57, 2.23)	<0.0001
Missing	399(0.2)	182(45.6)	94(23.6)	100(25.1)	2(0.5)	21(5.3)				

DRS: diabetes retinal screening

†had at least one event attended or booked

‡ Log binomial regression to test for an association with i) never attending retinal screening or ophthalmology and ii) attending retinal screening but not ophthalmology when referred, adjusting for all other variables in the table

Overall, 3,801 people (1.5%) had at least one retinal screening or ophthalmology appointment scheduled during the study period but attended none, while a further 2,039 (0.8%) attended retinal screening but not ophthalmology when referred (Table 1). All social variables explored were significantly associated with not attending diabetes eye care appointments. In multivariate log-binomial regression, men were more likely than women (RR 1.16 95%CI 1.08–1.23), and people aged 15–29 years were more likely than all other age groups to have never attended. Compared to NZ Europeans, Māori were more than twice as likely (RR 2.12 95%CI 1.95–2.31) and Pacific people were 1.41 times (95%CI 1.28–1.55) more likely to have not attended any appointments during the study period. The likelihood of attending no appointments increased with increasing deprivation (Table 1). The distributions of those attending retinal screening but not attending ophthalmology when referred followed a similar pattern for ethnicity and area level deprivation, though the effect sizes tended to be reduced (Table 1).

Among people who attended at least one retinal screening appointment between 1 July 2006 and 1 January 2018, lived at least 24 months from the date of the first screening appointment and were not immediately referred to ophthalmology on the first screening attendance (n = 181,265), the median follow-up time was 5.5 years (IQR 2.3–8.7) and the median number of retinal screening events attended was 3 (IQR 1–4). At the aggregate level, 62.1% of eligible people attended biennial screening (Table 2). After adjusting for sex, age, ethnicity and area-level deprivation, the groups less likely to access care every two years were people aged 70 years and above compared to those aged 50–59 years (70–79 years, RR = 0.87 95%CI 0.86–0.89 and ≥80 years, RR = 0.81 95%CI 0.78–0.83), and Māori and Pacific people compared to NZ Europeans (RR for both = 0.92 95%CI 0.91–0.93) (Table 2). The sensitivity analysis showed that this attendance rate increased to 77.3% for triennial screening (n = 168,622), with disparities being greater than those observed for biennial screening (S1 Table).

**Table 2:** Attendance at biennial retinal screening for diabetes, New Zealand, 2006–2019.

	Total†	Attendance at biennial screening		
	n (col%)	n (row%)	Relative Risk	p-value‡
**Total**	**181265(100)**	**112484(62.1)**		
**Sex**				
Female	85128(47.0)	51775(60.8)	Ref	
Male	96137(53.0)	60709(63.1)	1.03(1.02, 1.04)	<0.0001
**Age (years)**				
15–29	10483(5.8)	6193(59.1)	0.93(0.92, 0.95)	<0.0001
30–39	20532(11.3)	12454(60.7)	0.95(0.94, 0.97)	<0.0001
40–49	38257(21.1)	24298(63.5)	0.99(0.98, 1.00)	0.0806
50–59	46291(25.5)	29882(64.6)	Ref	
60–69	39212(21.6)	24763(63.2)	0.97(0.96, 0.98)	<0.0001
70–79	21670(12.0)	12354(57.0)	0.87(0.86, 0.89)	<0.0001
80+	4820(2.7)	2540(52.7)	0.81(0.78, 0.83)	<0.0001
**Ethnicity**				
NZ European	97556(53.8)	61410(62.9)	Ref	
Māori	26387(14.6)	15587(59.1)	0.92(0.91, 0.93)	<0.0001
Pacific	25790(14.2)	15214(59.0)	0.92(0.91, 0.93)	<0.0001
Asian	27454(15.1)	17912(65.2)	1.02(1.01, 1.03)	0.0006
Others	4078(2.2)	2361(57.9)	0.91(0.88, 0.93)	<0.0001
**Area level deprivation (quintiles)**				
Least deprived	24795(13.7)	15705(63.3)	Ref	
2	28637(15.8)	17825(62.2)	0.99(0.98, 1.00)	0.1486
3	33135(18.3)	20809(62.8)	1.00(0.99, 1.02)	0.5500
4	40554(22.4)	25177(62.1)	0.99(0.99, 1.01)	0.7757
Most deprived	53883(29.7)	32881(61.0)	0.99(0.99, 1.01)	0.8419
Missing	261(0.1)	87(33.3)		

† People who had at least one retinal screening appointment between 1 July 2006 and 1 January 2018, lived at least 24 months from the date of the first screening appointment and were not referred to ophthalmology on the same date as their first screening attendance

‡ Log binomial regression to test for an association with attending all biennial appointments, adjusting for all other variables in the table.

**Fig 2:**
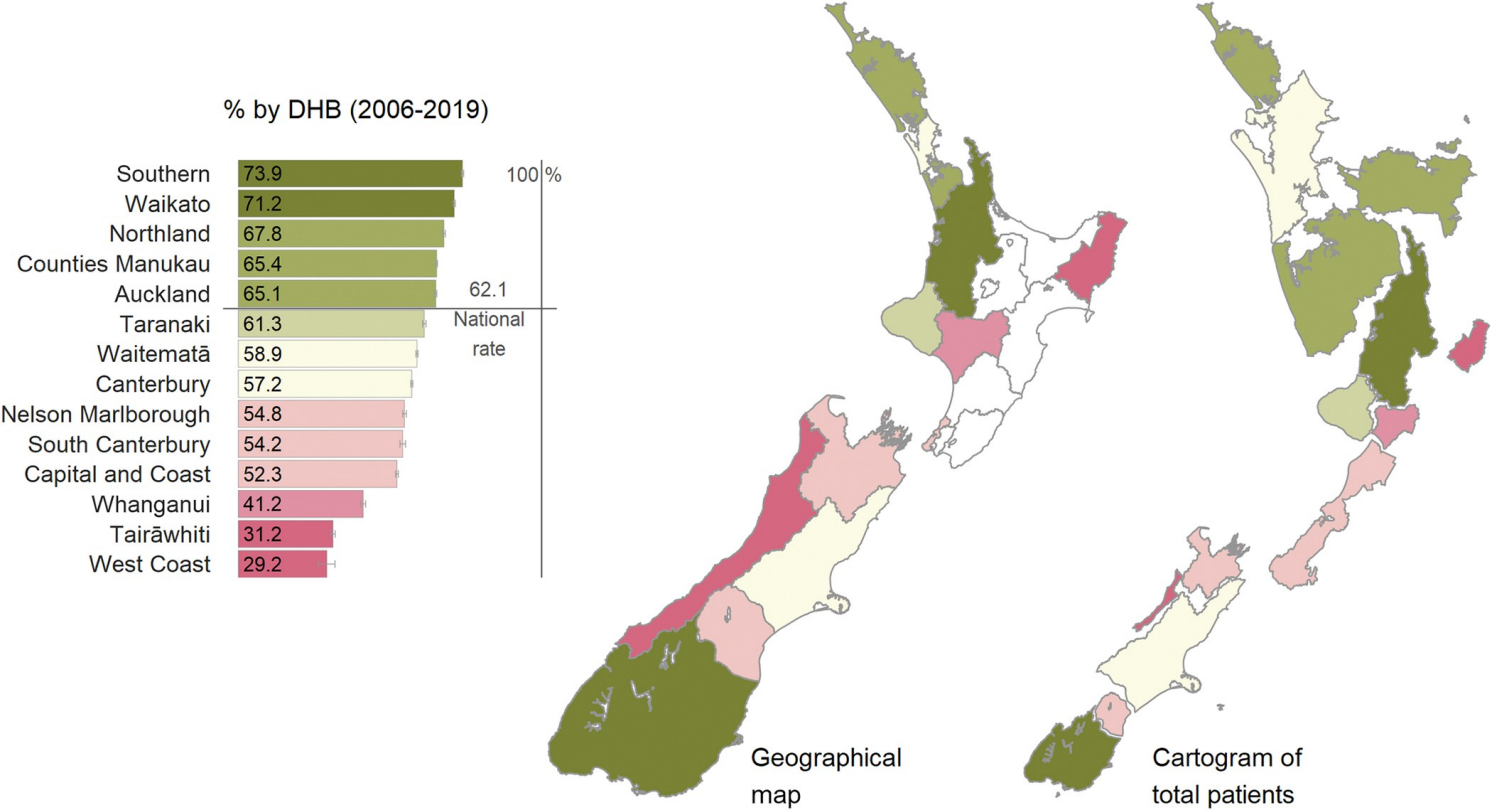
Rate of biennial screening by District Health Board (DHB) in New Zealand 2006–2019. **Note:** Incomplete data for six District Health Boards (DHBs) (Bay of Plenty, Hawke’s Bay, Lakes, Hutt Valley, Wairarapa, and Midcentral) meant these DHBs were omitted from analysis; the values displayed are unadjusted rates.

The biennial screening attendance rate showed a distinct geographical pattern (Fig 2). Generally, rates were higher than the national mean rate (62.1%) in northern and southern DHBs (green shades in Fig 2), while rates were lower in the more central DHBs (pink shades in Fig 2). The relatively high attendance rate in Auckland DHB is more evident in the cartogram compared to the geographical map due to the size of the DHB areas being proportionate to the number of total patients. The DHB with the greatest number of patients, Counties Manukau, had a rate slightly higher than the national rate. The rates in the West Coast and Tairāwhiti DHBs were strikingly low (<35%). However, the cartogram shows the number of patients impacted by these low rates was relatively small.

Further, access was consistently lower among Māori and Pacific peoples compared to NZ Europeans within each of the DHBs (S2 Table). The median disparity compared to NZ Europeans across DHBs was -5% for Māori and -4% for Pacific peoples, with disparities being largest in the major cities of Auckland and Wellington (Capital & Coast DHB), being -8% and -7% for Māori and -9% and -10% for Pacific peoples respectively). Disparities by age group were also varied across DHBs. For example, compared to rates among people aged 50–59 years, the median disparity with those aged 70–79 years and ≥80 years was -5% and -11% respectively at the national level, but were as large as -26% and -44% in South Canterbury DHB (S3 Table).

During the study period, 19,293 people underwent at least one laser treatment (being 7.8% of the study population and 16.5% of those who had at least one ophthalmology appointment) and 7,512 people had at least one anti-VEGF treatment event (being 3.1% of the study population and 6.4% of those having at least one ophthalmology appointment). Overall, 23,072 people had at least one laser or anti-VEGF treatment event between 2006 and 2019 (9.4% of the study population and 19.7% of those having at least one ophthalmology appointment).

People who were provided laser treatment for proliferative diabetic retinopathy received a median number of 1 laser treatment per person (IQR 1–3) over the study period. The likelihood of receiving at least one laser treatment was higher for women than men, for older compared to younger people, for all ethnicity groups compared to NZ Europeans—being highest among Pacific people—and increased with increasing deprivation (Table 3).

**Table 3:** Treatment with laser among New Zealanders presenting to at least one diabetes eye care event, 2006–2019.

Population subgroup	Total†	Laser Treatment (Attendance)		
	n (col%)	n (row%)	Relative Risk of treatment	p-value‡
**Total**	**245844(100)**	**19293(7.8)**		
**Sex**				
Female	116292(47.3)	9488(8.2)	Ref	
Male	129550(52.7)	9805(7.6)	0.95(0.93, 0.98)	<0.0001
Non-binary	2(0.0)	0(0.0)		
**Age (years)**				
15–29	15938(6.5)	719(4.5)	0.50(0.46, 0.54)	<0.0001
30–39	27579(11.2)	1473(5.3)	0.59(0.56, 0.63)	<0.0001
40–49	48526(19.7)	3450(7.1)	0.81(0.78, 0.85)	<0.0001
50–59	58721(23.9)	4899(8.3)	Ref	
60–69	51980(21.1)	4961(9.5)	1.20(1.16, 1.25)	<0.0001
70–79	32976(13.4)	3126(9.5)	1.26(1.21, 1.32)	<0.0001
80+	10124(4.1)	665(6.6)	0.91(0.84, 0.99)	0.0257
**Ethnicity**				
NZ European	132907(54.1)	9282(7.0)	Ref	
Māori	35176(14.3)	2453(7.0)	1.06(1.01, 1.11)	0.0103
Pacific	35216(14.3)	3896(11.1)	1.68(1.61, 1.75)	<0.0001
Asian	36795(15.0)	3238(8.8)	1.42(1.37, 1.48)	<0.0001
Others	5750(2.3)	424(7.4)	1.16(1.06, 1.28)	0.0017
**Area level deprivation (quintiles)**				
1 Least deprived	32960(13.4)	2054(6.2)	Ref	
2	38658(15.7)	2893(7.5)	1.17(1.11, 1.23)	<0.0001
3	44848(18.2)	3366(7.5)	1.19(1.12, 1.25)	<0.0001
4	55212(22.5)	4092(7.4)	1.16(1.10, 1.22)	<0.0001
5 Most deprived	73767(30.0)	6850(9.3)	1.38(1.31, 1.45)	<0.0001
Missing	399(0.2)	38(9.5)		

†had at least one retinal screening or ophthalmology event attended or booked

‡ Log binomial regression to test for an association with laser treatment, adjusting for all other variables in the table.

People who attended anti-VEGF treatment for diabetic maculopathy, (n = 7,512) attended a total of 52,576 appointments during the period 2014 (when it became available) to 2019. The median number of total injections per person was 4 (IQR 2–9). When the analysis was limited to the number of injections per person in their first year of treatment (n = 28,863 injections), the median per person was 3 (IQR 1–6); Māori were the ethnicity group with the fewest injections in the first year of treatment (2; IQR 1–4). There was considerable variation across DHBs, from 2 (IQR 1–4) in Southern to 6 (IQR 3–9) in Nelson Marlborough (S4 Table).

## Discussion

This study provides New Zealand’s first national-level estimates of diabetes eye service use among people aged ≥15 years, including attendance at retinal screening and ophthalmology, biennial screening rate and treatment with laser and anti-VEGF. Of people eligible to attend retinal screening, just under two-thirds received biennial screening (62.1%) and just over three-quarters (77.3%) attended at least once every three years. Further, we identified that almost 1 in 10 people (9.4%) who accessed any diabetes eye care during the study period required treatment via laser or anti-VEGF.

The biennial screening rate in our study (62.1%) was slightly higher than a recent estimate among adults ≥45 years in New South Wales, Australia (50.2%) [24], but lower than a study in the United States (71.1%) [25] that used comparable definitions. The period of follow-up may have influenced these results, being shorter in the US (4.8 years) study compared to our study (5.5 years) and the Australian study (11 years), giving fewer cycles requiring adherence. The World Health Organization recently included a measure of retinal screening coverage among people with diabetes as a core indicator for eye health [26], which may lead to more comparable data being reported in future.

We identified substantial variation between DHBs in terms of access to retinal screening, with three DHBs reaching fewer than 1 in 2 people with biennial screening (Fig 2). The range of models being implemented across the country have not been documented, but it appears strategies such as the screening team visiting well-distributed community-based sites as used in Northland [8] may help to achieve the relatively high rate of biennial screening (67.8%) observed there.

We confirmed findings from other studies in New Zealand [8, 14, 27] demonstrating inequities in the receipt of eye care by ethnicity. Compared with NZ Europeans, Māori were approximately twice as likely to never receive diabetes eye care or to access ophthalmology when referred (Table 1), 10% relatively less likely to receive biennial screening (Table 2) and received the fewest anti-VEGF injections when treatment was commenced. Similar findings were reported for Indigenous Australians, where retinal screening was less accessible to Indigenous compared to non-Indigenous Australians [28], suggesting systemic issues in eye services for Indigenous peoples with diabetes. The inequities in access to and outcomes of health care services experienced by Māori have been documented in academic as well as government documents, including the recent health and disability system review report [14, 16, 17]. In general, government strategies and guidelines aim to address these inequities. However, evidence specific to the diabetic eye care services is limited. Our analysis highlights a substantial gap in data availability for monitoring diabetes eye care in New Zealand. First, there is no register of people with diabetes. The Virtual Diabetes Register provides algorithm-based estimates of the prevalence of diabetes [7] but it is not recommended to be used to inform individual level clinical interventions since it could include people incorrectly identified as having diabetes when they do not [7, 29]. This lack of national diabetes registry severely limits its analytical capacity. For example, we accessed the Virtual Diabetes Register dataset to estimate the national retinal screening service use but could not use it to form the denominator of our analysis because individuals moved on and off the Register over the study period. If a diabetes register existed, it would allow an assessment of the extent to which all people with diabetes have accessed eye care (population coverage), rather than those who had a diabetes eye care appointment scheduled.

We found 1.5% of people had never accessed care following at least one scheduled appointment, which is comparable to that reported by a US study, where 1.4% of people had no screening during the 4.8 years of follow-up [25] and an Australian study, where 1.0% had no optometry or ophthalmology claims over at least 5 years of follow-up) [24]. However, there may be a much larger group of people with diabetes in New Zealand who have never been referred or had an appointment scheduled. Our estimate of people unable to access care is therefore likely an underestimate. Indeed, a National Eye Health Survey in Australia (2015/16) reported that 15.3% of non-Indigenous and 26.2% of Indigenous Australians aged ≥40years with self-reported diabetes had never had a diabetic eye examination [28]. Similarly, a multi-country survey conducted in Germany, Saudi Arabia, Japan, Romania, Mexico, Argentina, Uganda and Bangladesh in 2017 reported that 21% of the adults with diabetes had never had an eye examination for diabetic eye disease [30]. Having an accurate register of people with diabetes would greatly enhance management of diabetes and is essential to rule out disparities in access to care.

The data currently reported to the Ministry of Health are limited to counting publicly funded diabetes eye care service events (as reported here), and not the details or outcomes of these services. While additional diabetes eye care services may be provided by the private sector this is likely to be for a relatively small number of individuals and would require further investigation. The lack of outcome data means there is no readily available way to monitor the quality and effectiveness of diabetes eye care. For example, we were unable to distinguish any clinical reason why people may have not maintained attendance at biennial screening, such as referral for cataract surgery. We were also unable to report the extent of vision loss from DR, the key outcome for diabetes eye care services, the extent to which people requiring more regular screening (<24 months) received it, or the extent to which people requiring treatment received it. We found an average of three anti-VEGF treatments per person accessed in the first year of treatment, compared to six or seven treatments in a recent prospective study of 220 patients in Australia and New Zealand (conducted in Auckland DHB) [31]. The treatment rates of anti-VEGF we calculated likely under-estimate the amount of treatment needed, but more detailed analysis is required to ascertain whether it reflects lack of uptake of an offered treatment or a rationing of treatment offered by the DHB—Auckland does not ration anti-VEGF treatment but other DHBs do, which led to wide variation in treatment (S4 Table).

We recommend strategies to both identify and address the structural determinants and health system factors, including DR data infrastructure and monitoring systems that exist in diabetes eye care services in order to develop pathways that will eliminate these inequities [32, 33]. In addition to the ethnic disparities observed, we found that younger adults accessed services less than other age groups. Given the growing prevalence of Type 2 diabetes among younger New Zealanders [34–37] and the longer time these people live with diabetes and its potential complications, it is essential that future work prioritises the development of eye care services that meet the needs of young people with diabetes.

This analysis must be interpreted in the context of several limitations. The Ministry of Health had no or incomplete data for almost one-third of the DHBs which precluded their inclusion in our analysis. These omitted DHBs represent approximately 15.0% of people ≥15 years living with diabetes in 2019 [7]. This relatively small proportion of the population with diabetes means our results are broadly generalisable nationally. However, the omission of these DHBs may have led to under-estimates of our disparities across ethnicity groups and area level deprivation—the DHBs with missing data had a higher proportion of the population who were Māori (21.2% compared to 15.1% of our sample) and in the most deprived quintile (34.7% compared to 30.6%).

A further limitation is that we analysed ethnicity grouped into the five level 1 categories of Statistics New Zealand—Māori, NZ European, Pacific, Asian and Others. We recognise there is large heterogeneity within these categories in relation to diabetes, particularly people of Asian ethnicity, with a much higher prevalence among South Asians compared to East Asians [38, 39].

Overall, our analysis has identified considerable issues with access to diabetes eye care in New Zealand, and inequity across DHBs, ethnicity groups and area level deprivation. In 2022 the government commenced major health sector reform that will see increased focus on improving quality, consistency and equity of care delivered through a single nationwide health service [40]. This reform provides an opportunity to redesign a national diabetes eye care program that is flexible to meet the needs of local communities, delivers equity and is appropriately funded. Our analysis has highlighted that strengthening data collection and monitoring must form part of any service improvement, including a diabetes register and better data linkage between retinal screening and ophthalmology clinics [41]. These data can then inform efforts to improve access to—and quality of—diabetes eye care services for all New Zealanders.

## Supporting information

S1 Table Sensitivity analysis for retinal screening attendance rate once every 3 years, New Zealand, 2006–2019. (PDF)

S2 Table Heat map of disparities in biennial screening rate by ethnicity, across District Health Boards, New Zealand 2006–2019. (PDF)

S3 Table Heat map of disparities in biennial screening rate by age group, across District Health Boards, New Zealand 2006–2019. (PDF)

S4 Table Heat map of the number of anti-VEGF injections in the first year of treatment (within 365 days from the date of the first dose) among people receiving anti-VEGF treatment across District Health Boards, New Zealand, 2014–2019. (PDF)
