# Supplementary material for: Use of public sector diabetes eye services in New Zealand 2006–2019: Analysis of national routinely collected datasets
Source: PLoS One. 2023 May 18;18(5):e0285904. doi: 10.1371/journal.pone.0285904 (PMC10194990; doi:10.1371/journal.pone.0285904)
Supplement: S1 Table — (PDF) [file pone.0285904.s001.pdf]

S1 Table: Sensitivity analysis for retinal screening attendance rate once every 3 years, New Zealand, 2006–2019

	**Total†**	**Attendance at retinal screening every three years**		
	n (col%)	n (row%)	Odds Ratio‡	p-value
**Total**	**168622(100)**	**130331(77.3)**		
**Sex**				
Female	79588(47.2)	60474(76.0)	Ref	
Male	89034(52.8)	69857(78.5)	1.1(1.07 , 1.12)	<0.0001
**Age (years)**				
15-29	9143(5.4)	6732(73.6)	0.72(0.68 , 0.76)	<0.0001
30-39	18570(11.0)	14299(77.0)	0.87(0.83 , 0.90)	<0.0001
40-49	35514(21.1)	28408(80.0)	1.01(0.98 , 1.05)	0.4930
50-59	43701(25.9)	35142(80.4)	Ref	
60-69	37265(22.1)	28820(77.3)	0.80(0.77 , 0.83)	<0.0001
70-79	20219(12.0)	14231(70.4)	0.53(0.51 , 0.55)	<0.0001
80+	4210(2.5)	2699(64.1)	0.39(0.37 , 0.42)	<0.0001
**Ethnicity**				
NZ European	90966(53.9)	71549(78.7)	Ref	
Māori	24299(14.4)	18314(75.4)	0.76(0.74 , 0.79)	<0.0001
Pacific	24151(14.3)	17742(73.5)	0.69(0.66 , 0.71)	<0.0001
Asian	25415(15.1)	19998(78.7)	0.89(0.86 , 0.92)	<0.0001
Others	3791(2.2)	2728(72.0)	0.63(0.59 , 0.68)	<0.0001
**Area level deprivation (quintiles)**				
Least deprived	23044(13.7)	18480(80.2)	Ref	
2	26675(15.8)	20857(78.2)	0.91(0.87 , 0.95)	<0.0001
3	30830(18.3)	23865(77.4)	0.88(0.85 , 0.92)	<0.0001
4	37772(22.4)	29252(77.4)	0.91(0.87 , 0.94)	<0.0001
Most deprived	50044(29.7)	37754(75.4)	0.84(0.81 , 0.88)	<0.0001
Missing	257(0.2)	123(47.9)		

† People who had at least retinal screening appointment between 1 July 2006 and 1 January 2017, lived at least 36 months from the date of the first screening appointment and were not immediately referred to ophthalmology on the first screening attendance

‡ Logistic regression to test for an association with attendance at retinal screening every three years, adjusting for all other variables in the table.
