# Supplementary material for: Use of public sector diabetes eye services in New Zealand 2006–2019: Analysis of national routinely collected datasets
Source: PLoS One. 2023 May 18;18(5):e0285904. doi: 10.1371/journal.pone.0285904 (PMC10194990; doi:10.1371/journal.pone.0285904)
Supplement: S2 Table — (PDF) [file pone.0285904.s002.pdf]

S2 Table: Heat map of disparities in biennial screening rate by ethnicity, across District Health Boards, New Zealand 2006 – 2019

District Health Board	Disparity with NZ European rate				NZ European rate	Reference: Total eligible population for biennial screening by ethnicity					Number of eligible individuals attending biennial screening by ethnicity				
	Asian	Māori	Pacific	Others		Asian	Māori	Pacific	Others	NZ European	Asian	Māori	Pacific	Others	NZ European
Southern	4	-5	-7	-6	74	418	992	385	144	11661	327	686	261	98	8679
Waikato	0	-6	-6	-8	73	1651	5276	967	558	12769	1207	3527	650	366	9366
Counties Manukau	0	-7	-8	-7	69	8008	4702	12458	932	8158	5561	2950	7636	583	5669
Auckland	-3	-8	-9	-12	69	6938	1535	4821	561	5702	4617	932	2914	323	3945
Northland	6	-2	0	-12	69	226	4600	177	60	5625	168	3055	121	34	3872
Taranaki	3	-8	-3	-9	63	169	1116	85	80	5847	111	606	51	43	3663
Waitematā	2	-3	-4	-7	60	5846	1907	3541	684	11668	3573	1084	1972	359	6950
Canterbury	2	-4	-1	-7	57	1739	1537	930	452	16171	1040	821	525	227	9292
Nelson Marlborough	-3	-6	1	10	55	156	529	107	75	5708	81	262	60	49	3154
Capital and Coast	-1	-7	-10	-1	55	2078	1279	2060	416	6395	1117	609	927	225	3522
South Canterbury	19	2	-5	-2	54	52	162	35	44	2703	38	90	17	23	1455
Whanganui	1	-1	-4	12	41	96	950	83	30	2640	41	382	31	16	1095
Tairāwhiti	14	3	9	.	30	48	1726	95	25	2143	21	559	37	7	636
West Coast	.	4	.	.	28	1	34	3	4	246	1	11	0	3	69
**National**	**2**	**-4**	**-4**	**-5**	**63**	**27426**	**26345**	**25747**	**4065**	**97436**	**17903**	**15574**	**15202**	**2356**	**61367**
Median disparities across DHBs	2	-5	-4	-7											

Note: Incomplete data for six District Health Boards (DHBs) (Bay of Plenty, Hawke's Bay, Lakes, Hutt Valley, Wairarapa, and Midcentral) meant these DHBs were omitted from analysis; the values displayed in the ethnicity columns are rate differences (unadjusted) from the European rate (e.g., Asian = 1% means (Asian rate [%] – European rate [%] = 1%); no values are displayed if there was fewer than 30 eligible people in an ethnicity group. Records of people where the DHB is unspecified were excluded (n=236).
