# Supplementary material for: Use of public sector diabetes eye services in New Zealand 2006–2019: Analysis of national routinely collected datasets
Source: PLoS One. 2023 May 18;18(5):e0285904. doi: 10.1371/journal.pone.0285904 (PMC10194990; doi:10.1371/journal.pone.0285904)
Supplement: S3 Table — (PDF) [file pone.0285904.s003.pdf]

S3 Table: Heat map of disparities in biennial screening rate by age group, across District Health Board, New Zealand 2006 – 2019

District Health Board	Disparity with 50-59 years group						50-59 age group rate	Total eligible population for biennial screening (years)							Number of eligible individuals attending biennial screening (years)						
	15-29	30-39	40-49	60-69	70-79	80+		15-29	30-39	40-49	60-69	70-79	80+	50-59	15-29	30-39	40-49	60-69	70-79	80+	50-59
Southern	-8	-5	-1	-3	-7	-15	77	635	1108	2370	3439	2178	563	3307	440	798	1812	2566	1530	350	2555
Waikato	-10	-6	-3	1	-3	-11	74	1315	2308	4176	4651	2714	671	5386	843	1554	2931	3474	1924	423	3967
Northland	-7	-4	-1	-1	-10	-21	71	472	995	2153	2539	1324	249	2956	301	663	1495	1774	806	124	2087
Counties Manukau	-11	-7	-2	-5	-14	-26	70	2467	5256	9166	5926	2122	310	9011	1451	3273	6218	3862	1175	135	6285
Auckland	-4	-3	0	-5	-12	-16	68	1236	2471	4556	3887	1929	391	5087	790	1614	3121	2446	1085	205	3470
Taranaki	-14	-7	-2	1	-3	-5	63	230	549	1177	1794	1284	438	1825	113	310	720	1156	768	254	1153
Waitematā	-2	-2	1	-2	-14	-18	61	1445	2921	5311	4995	2507	441	6026	860	1746	3302	2957	1179	190	3704
South Canterbury	2	3	0	-3	-26	-44	61	126	218	472	817	528	86	749	79	139	285	471	181	14	454
Nelson Marlborough	-4	-1	-1	0	-5	-10	57	238	450	1009	1692	1185	320	1681	126	251	562	955	612	149	951
Canterbury	1	-1	-3	3	4	4	56	1196	2123	3752	4837	3121	808	4992	682	1171	2006	2880	1879	487	2800
Capital and Coast	-5	-6	-5	1	-2	-10	55	757	1399	2652	2682	1454	243	3041	375	684	1318	1492	761	109	1661
Whanganui	2	-5	-2	4	1	1	41	138	335	666	888	594	141	1037	59	121	261	398	245	59	422
Tairāwhiti	0	1	1	-2	-4	-7	32	187	338	692	950	689	153	1028	60	112	233	292	192	39	332
West Coast	.	.	12	13	.	.	20	13	29	59	65	16	3	103	6	10	19	22	5	1	21
**National**	-5	-4	-1	-1	-8	-12	65	**10455**	**20500**	**38211**	**39162**	**21645**	**4817**	**46229**	**6185**	**12446**	**24283**	**24745**	**12342**	**2539**	**29862**
Median disparity across DHBs	-4	-4	-1	0	-5	-11															

Note: Incomplete data for six District Health Boards (DHBs) (Bay of Plenty, Hawke's Bay, Lakes, Hutt Valley, Wairarapa, and Midcentral) meant these DHBs were omitted from analysis; the values displayed in the age group columns are rate differences (unadjusted) from the rate for those aged 50-59 years; no values are displayed if there was fewer than 30 eligible people in an age group.

Records of people where the DHB was unspecified were excluded from analysis (n=236).
