# Supplementary material for: Use of public sector diabetes eye services in New Zealand 2006–2019: Analysis of national routinely collected datasets
Source: PLoS One. 2023 May 18;18(5):e0285904. doi: 10.1371/journal.pone.0285904 (PMC10194990; doi:10.1371/journal.pone.0285904)
Supplement: S4 Table — (PDF) [file pone.0285904.s004.pdf]

S4 Table: Heat map of the number of anti-VEGF injections in the first year of treatment (within 365 days from the date of the first dose) among people receiving anti-VEGF treatment across District Health Boards, New Zealand, 2014-2019

District Health Board	Injections per person in first year of treatment (n)			Total number of people treated	Total number of injections
	Median	Inter-quartile range			
		Q1	Q3		
Nelson Marlborough	6	3	9	291	1773
Canterbury	4	2	7	850	3980
Waitematā	4	2	6	908	3952
Auckland	3	2	6	842	3222
Capital and Coast	3	2	5	392	1395
Counties Manukau	3	1	5	2071	7482
South Canterbury	3	2	5	133	523
Tairāwhiti	3	1	4	117	353
Taranaki	3	1	4	236	754
Waikato	3	1	5	505	1845
West Coast	3	1	6	75	304
Whanganui	3	2	4	46	137
Northland	2	1	5	308	967
Southern	2	1	4	733	2167
Unknown	1	1	3	5	9
**National**	**3**	**1**	**6**	**7512**	**28863**

Note: Incomplete data for six District Health Boards (DHBs) (Bay of Plenty, Hawke's Bay, Lakes, Hutt Valley, Wairarapa, and Midcentral) meant these DHBs were omitted from analysis
